# Contract labor costs and financial performance at high and low surgical volume hospitals

**DOI:** 10.1371/journal.pone.0356994

**Published:** 2026-08-27

**Authors:** Samuel J. Enumah, Xue Wu, Xueya Cai, Yue Li

**Affiliations:** 1 Department of Surgery, University of Rochester Medical Center, Rochester, New York, United States of America; 2 Department of Public Health Sciences, University of Rochester Medical Center, Rochester, New York, United States of America; 3 Surgical Health Outcomes Research Enterprise, University of Rochester Medical Center, Rochester, New York, United States of America; 4 Department of Biostatistics and Computational Biology, University of Rochester Medical Center, Rochester, New York, United States of America; University of Colorado - Anschutz Medical Campus, UNITED STATES OF AMERICA

## Abstract

**Background:**

Rising labor costs negatively impact hospitals’ financial stability. Poor hospital financial performance raises the probability of bankruptcy and closure, which limits patients’ access to critically important medical and surgical care. The objective of the study was to determine the association between contract labor costs and hospital financial performance and examine if this relationship is modified by surgical volume.

**Methods:**

We used data from the National Academy for State Health Policy, the American Hospital Association, and the RAND Corporation to analyze general and financial characteristics of U.S. hospitals. The primary outcomes were hospital operating margin, total margin, and financial distress. Generalized estimating equations were used to identify associations between contract labor costs and financial performance.

**Results:**

Our sample included 22,366 hospital-year observations from 2,914 hospitals. Higher contract labor costs were associated with lower operating margins (coefficient −0.13, 95% confidence interval (CI) −0.17 to −0.10; p < 0.001), lower total margins (coefficient −0.11, 95% CI −0.14 to −0.07; p < 0.001), and higher financial distress (odds ratio 1.01, 95% CI 1.00 to 1.02; p = 0.003). High surgical volume was associated with higher operating margins (coefficient 1.40, 95% CI 0.86 to 1.95; p < 0.001), higher total margins (coefficient 1.62, 95% CI 1.12 to 2.12; p < 0.001), and a lower risk of financial distress (OR 0.77, 95% CI 0.64 to 0.94; p = 0.008) compared to low surgical volume. The interaction term for contract labor costs and high-volume status was not statistically significant for operating margin (coefficient −0.02, 95% CI −0.08 to 0.05; p = 0.605), total margin (coefficient −0.04, 95% CI −0.09 to 0.02; p = 0.179), or financial distress (OR 1.01, 95% CI 0.99 to 1.03; p = 0.303).

**Conclusions:**

Higher contract labor costs were associated with lower hospital profit margins. High surgical volume may not offer financial protection against the economic challenge of contract labor costs.

## Introduction

Healthcare delivery in the United States remains expensive, and the costs associated with providing healthcare continue to rise [1]. In 2023, hospital care spending reached $1.5 trillion—approximately one-third of the annual national health care expenditure—and hospital spending as a percent of gross domestic product is expected to rise from 5.5% in 2023 to 6.0% in 2032 [2]. Hospital care remains costly, and increases in the prices of labor, supplies, drugs, and technology contribute to rising hospital care expenditures.

Labor costs remain the largest contributor to hospital expenses. From 2021 to 2023, labor costs increased by $42.5 billion [3]. In 2024, labor costs accounted for $890 billion and represented 56% of overall hospital expenses [4]. Wages and benefits drive labor expenses, and staffing remains a critical problem facing hospital leadership [5]. Since the COVID-19 pandemic, hospitals and health systems have struggled with recruitment and retention, and the limited supply of labor has contributed to the increase in hospital expenses [6]. To address staff shortages, hospitals turned to contract labor—temporary staff—to fill the gaps in their workforce [7]. While rising contract labor costs remain a challenge for hospital leaders, the degree to which this expense affects financial performance is not well characterized. In addition, identifying a relationship between modifiable factors (e.g., surgical volume) and contract labor costs remains understudied.

To address these knowledge gaps, our study aimed to determine the association between contract labor costs and hospital financial performance and to identify if surgical volume moderates this relationship. We hypothesized that higher contract labor costs were associated with worse financial performance (lower financial margins and a higher risk of financial distress). In addition, we chose to explore the extent to which surgical volume may mitigate the financial challenges faced by hospitals. High-volume centers have high clinical demands and may need to hire temporary staff despite remarkably high labor price increases. As contract labor costs rise, high-volume surgical hospitals may be insensitive to prices and worse off compared to low-volume hospitals. However, high-volume hospitals hire contract labor to fuel profitable service lines, which include surgical care. Therefore, high-volume surgical hospitals may be somewhat shielded from the negative financial effect of rising contract labor costs as compared with low-volume hospitals. Given these possible financial implications of high surgical volume, we hypothesized that high surgical volume mitigates the negative association between contract labor costs and hospital financial performance. Therefore, the magnitude of the negative association between contract labor costs and hospital financial performance would be lower at high-volume hospitals compared to low-volume hospitals.

## Methods

### Data

We used hospital information from the National Academy for State Health Policy (NASHP) Hospital Cost Tool, the American Hospital Association (AHA), and the RAND Hospital Data for years 2011–2023. The NASHP Hospital Cost Tool is a publicly available dataset based on the Centers for Medicare & Medicaid (CMS) Cost Reports, and the tool was designed to provide hospitals and policymakers with essential information about hospital costs [8]. The AHA conducts an annual survey of more than 6,000 U.S. hospitals and 400 health systems, and the AHA database includes information about hospital utilization, organization, workforce, finances, and services [9]. The RAND Hospital Data organizes the CMS cost reports from the Healthcare Provider Cost Reporting Information System (HCRIS), and the dataset provides general information about hospital characteristics and detailed financial information including revenues, expenses, assets, and liabilities [10]. Hospital data from these three sources were merged based on the Medicare Provider Identification Number. We included short-term general and specialty hospitals. We excluded critical access hospitals as they have a different reimbursement structure (see S1 Fig for inclusions and exclusions).

### Variables

The main dependent variables were operating margin, total margin, and financial distress. Operating margin was defined as operating profit divided by operating revenue. Total margin was defined as net income divided by the sum of net patient revenue and all other income. Financial distress was defined based on the modified Altman Z-score, which captures multiple aspects of organizational financial performance including profitability, efficiency, liquidity, and leverage [11]. Previous literature identified that the modified Altman Z-score was effective in predicting U.S. hospital financial distress that resulted in bankruptcy [12]. We categorized a hospital as financially distressed if the modified Altman Z-score was less than 1.8 [12–15]. Full details regarding the modified Altman Z-score calculation are provided in the Supporting Information File (see **S1 Appendix**). We removed observations with negative liabilities, assets, and expenses or those with missing values in key variables. To avoid the influence of extreme outliers, the values for operating margin, total margin, and the Altman Z-score were trimmed at the 1^st^ and 99^th^ percentiles.

Our main independent variable was direct patient care contracted labor cost as a percentage of direct patient care labor cost (subsequently referred to as “contract labor costs”), and this was extracted from the NASHP dataset. Additionally, we extracted surgical volume from the AHA dataset. We defined high-volume hospitals (HVHs) as hospitals with a reported annual surgical volume at or above the 75^th^ percentile. We defined low-volume hospitals (LVHs) as hospitals whose surgical volume was below this threshold.

Other independent variables included hospital characteristics. Rural hospitals were defined as those located in rural or micropolitan core based statistical areas (CBSAs) [16]. Teaching hospitals included both major and minor teaching hospitals. Hospital bed capacity was grouped into three categories: low (1–99), medium (100–299), and high (300 or more). Ownership included the categories of non-profit, for-profit, and government. For market competition, we calculated the Herfindahl-Hirschman Index (HHI) based on hospital discharges [17]. The HHI was categorized as unconcentrated (0–1,499), moderately concentrated (1,500–2,499), or highly concentrated (2,500–10,000) [18]. Hospitals were categorized as members of a health system or independent. The wage index compares hospital workers’ mean hourly wage in each CBSA to the national mean wage [19]. The wage index captured differences in hospital wage rates among various labor markets. To account for different patient complexity across hospitals, we included case mix index, which represents the average relative diagnosis-related group weight of a hospital’s inpatient discharges [20]. A higher case mix index indicates that a hospital provides care for more complex and resource-intensive patients.

### Statistical analysis

Descriptive statistics were generated for the sample of hospitals. We used t-tests, Wilcoxon rank sum tests, and chi-square tests to compare means, medians, and proportions between high-volume and low-volume hospitals. For 2011–2023, the unadjusted average hospital operating margins, total margins, contract labor costs, and proportions of hospitals experiencing financial distress were reported for high-volume hospitals and low-volume hospitals.

To determine the independent associations between contract labor costs and financial performance, we fit three regression models. Given the hierarchical data structure with multiple hospital-year observations and given our interest in a population-average effect, we applied the generalized estimation equation (GEE) method to account for the within-hospital clustering effect [21]. Operating margin was modeled against contract labor costs in the GEE model, and the hospital covariates described above were included in the models. Given our interest in exploring annual surgical volume as a potential modifier, we included an interaction term of high-volume status and contract labor costs. The model also included year fixed effects, robust standard errors, and an exchangeable correlation structure to take into account the hospital clustering effect. We calculated the average adjusted predictions of the dependent variable at representative values of contract labor costs using the postestimation margins and marginsplot Stata commands. We estimated two additional GEEs with total margin and financial distress as dependent variables. For total margin, the identity link function was used. Given that financial distress is a binary variable, a logit link function and binomial distribution were used. Additional details regarding the model specification are included in the Supporting Information File (**S1 Appendix**).

We performed sensitivity analyses with alternative cutoffs for defining high-volume hospitals and with separate time frames for 2011−2019 versus 2020−2023 given the potential influence of COVID-19. Statistical significance was defined as a two-tailed p value less than 0.05. For the three outcome measures in the regression analyses, a Bonferroni correction was applied, and significance was defined as a p value less than 0.017. Coefficients, 95% confidence intervals (CI), and p values were reported. This study did not involve human subjects. Data analysis was performed between August 2025 and November 2025 using Stata, Version 19 (StataCorp LLC, College Station, TX). The Strengthening the Reporting of Observational Studies in Epidemiology (STROBE) reporting guideline was used [22].

## Results

Our sample included 22,366 hospital-year observations from 2,914 unique hospitals. In 2017, the median contract labor cost was 3.2% (interquartile range (IQR) 1.5–6.1, Table 1). The median hospital operating margin and total margin were 3.6% (IQR −1.5–9.3) and 4.2 (IQR −0.6–9.6) respectively. Financial distress was identified in 17.9% of hospitals. In 2017, 23.3% of hospitals were rural, 59.4% were teaching, 42.3% had 100–299 beds, 70.5% were non-profit, 56.2% were in unconcentrated markets, and 71.3% were part of a health system. The median annual surgical volume was 6,441 (IQR 3,433–11,813). In 2017, HVHs were more commonly teaching hospitals (92.9% vs. 48.2%, p < 0.001), large bed size (77.3% vs. 12.9%, p < 0.001), non-profit (78.7% vs. 67.8%, p < 0.001), and members of a health system (77.5% vs. 69.2%, p = 0.001) compared to LVHs. The median annual surgical volume was 17,470 (IQR 14,023–24,202) at HVHs and 4,866 (IQR 2,843–7,424) at LVHs. The median contract labor costs were lower at HVHs compared to LVHs (2.8% vs 3.4%, p = 0.004). At HVHs, the median operating margin was higher than at LVHs (5.4% vs. 2.9%, p < 0.001), and the median total margin was higher (6.2% vs. 3.3%, p < 0.001) compared to LVHs.

**Table 1:** Hospital characteristics at high and low volume hospitals in 2017.

Characteristics	All Hospitals (n = 1,741)	High Volume (n = 436)	Low Volume (n = 1,305)	P value
Rural, No. (%)	406 (23.3)	17 (3.9)	389 (29.8)	<.001
Teaching Hospital, No. (%)	1,034 (59.4)	405 (92.9)	629 (48.2)	<.001
Bed Size, No. (%)				
< 99	499 (28.7)	5 (1.2)	494 (37.9)	<.001
100–299	737 (42.3)	94 (21.6)	643 (49.3)	
> 300	505 (29.0)	337 (77.3)	168 (12.9)	
Ownership, No. (%)				
Non-profit	1,228 (70.5)	343 (78.7)	885 (67.8)	<.001
Government	270 (15.5)	67 (15.4)	203 (15.6)	
For-profit	243 (14.0)	26 (6.0)	217 (16.6)	
Herfindahl Hirschman Index, No. (%)				
Unconcentrated	978 (56.2)	244 (56.0)	734 (56.3)	0.239
Moderately Concentrated	358 (20.6)	80 (18.4)	278 (21.3)	
Highly Concentrated	405 (23.3)	112 (25.7)	293 (22.5)	
System Member, No. (%)	1,241 (71.3)	338 (77.5)	903 (69.2)	0.001
Wage Index, mean (SD)	0.98 (0.19)	1.01 (0.17)	0.97 (0.19)	<.001
Case Mix Index, mean (SD)	1.60 (0.32)	1.83 (0.25)	1.52 (0.30)	<.001
Contract Labor Costs (%), median (IQR)	3.2 (1.5 - 6.1)	2.8 (1.2 - 5.3)	3.4 (1.5 - 6.4)	0.004
Surgical Volume, median (IQR)	6,441 (3,433 − 11,813)	17,470 (14,023 - 24,202)	4,866 (2,843 − 7,424)	<.001
Outcomes				
Operating Margin (%), median (IQR)	3.6 (−1.5 - 9.3)	5.4 (1.4 - 9.9)	2.9 (−2.4 - 9.0)	<.001
Total Margin (%), median (IQR)	4.2 (−0.6 - 9.6)	6.2 (2.4 - 11.1)	3.3 (−1.7 - 9.2)	<.001
Financial Distress, No. (%)	311 (17.9)	52 (11.9)	259 (19.9)	<.001

Notes: Statistical tests compare high-volume hospitals and low-volume hospitals. Chi-squared test for Rural, Teaching hospitals, Bed size, Ownership, HHI, System member, and Financial Distress; T-test for Wage index and Case Mix Index; Wilcoxon rank-sum (Mann–Whitney) test for Operating margin, Total margin, Contract labor costs, and Surgical volume. Due to rounding, the combined percentages may exceed 100% for some variables.

**Fig 1:**
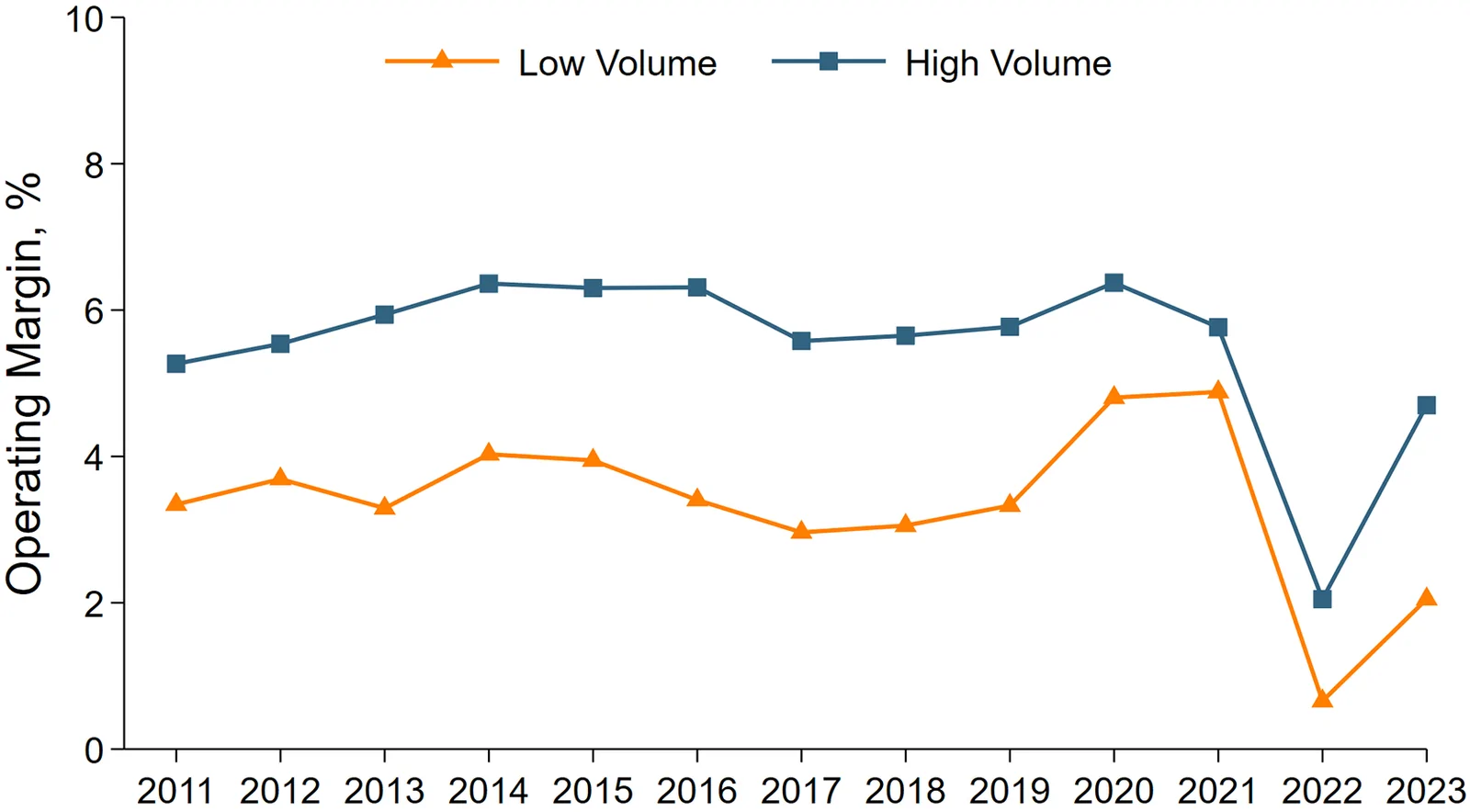
Average operating margins by surgical volume, 2011–2023. The figure presents the unadjusted average operating margin in each year for high-volume and low-volume hospitals.

**Fig 2:**
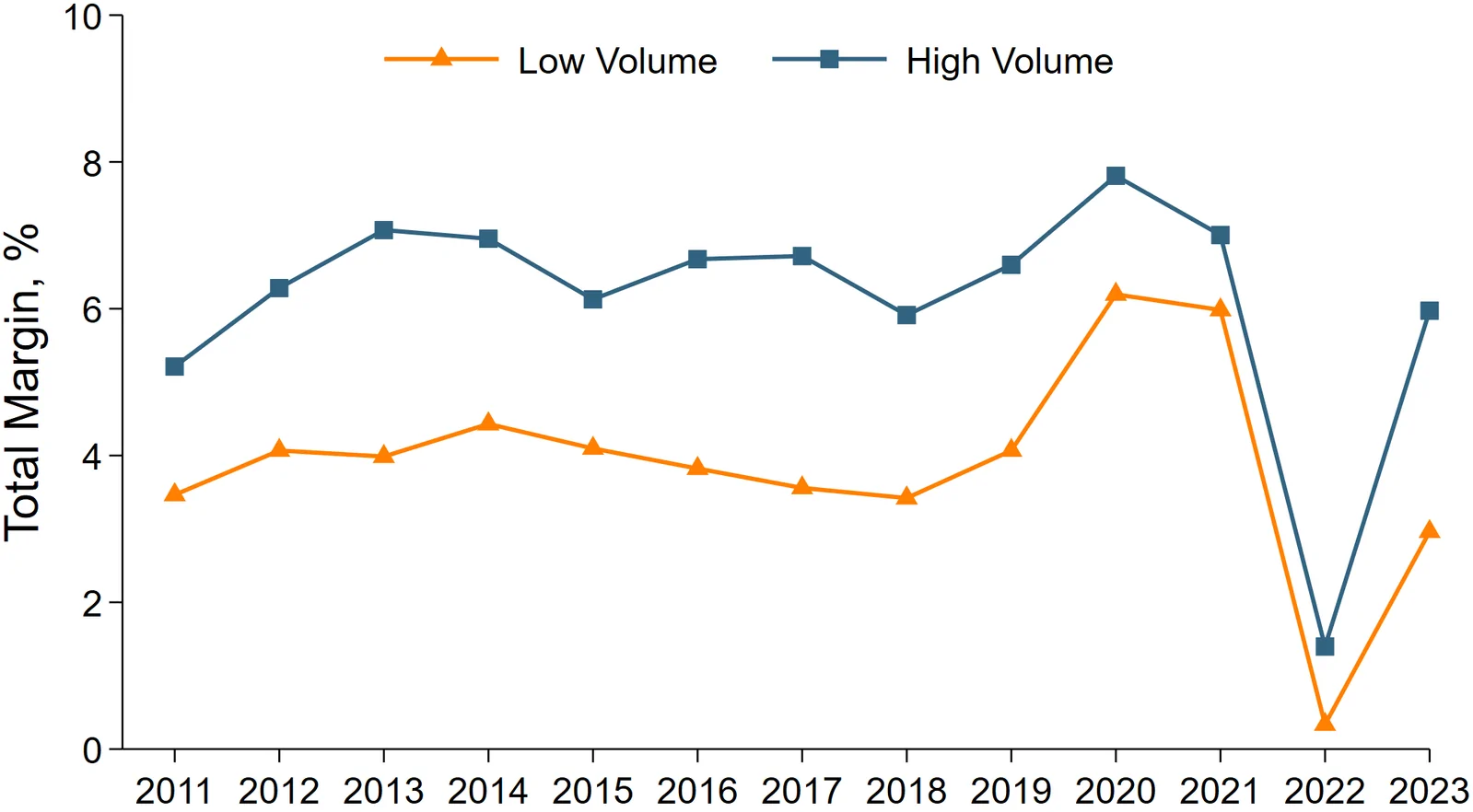
Average total margins by surgical volume, 2011–2023. The figure presents the unadjusted average total margin in each year for high-volume and low-volume hospitals.

**Fig 3:**
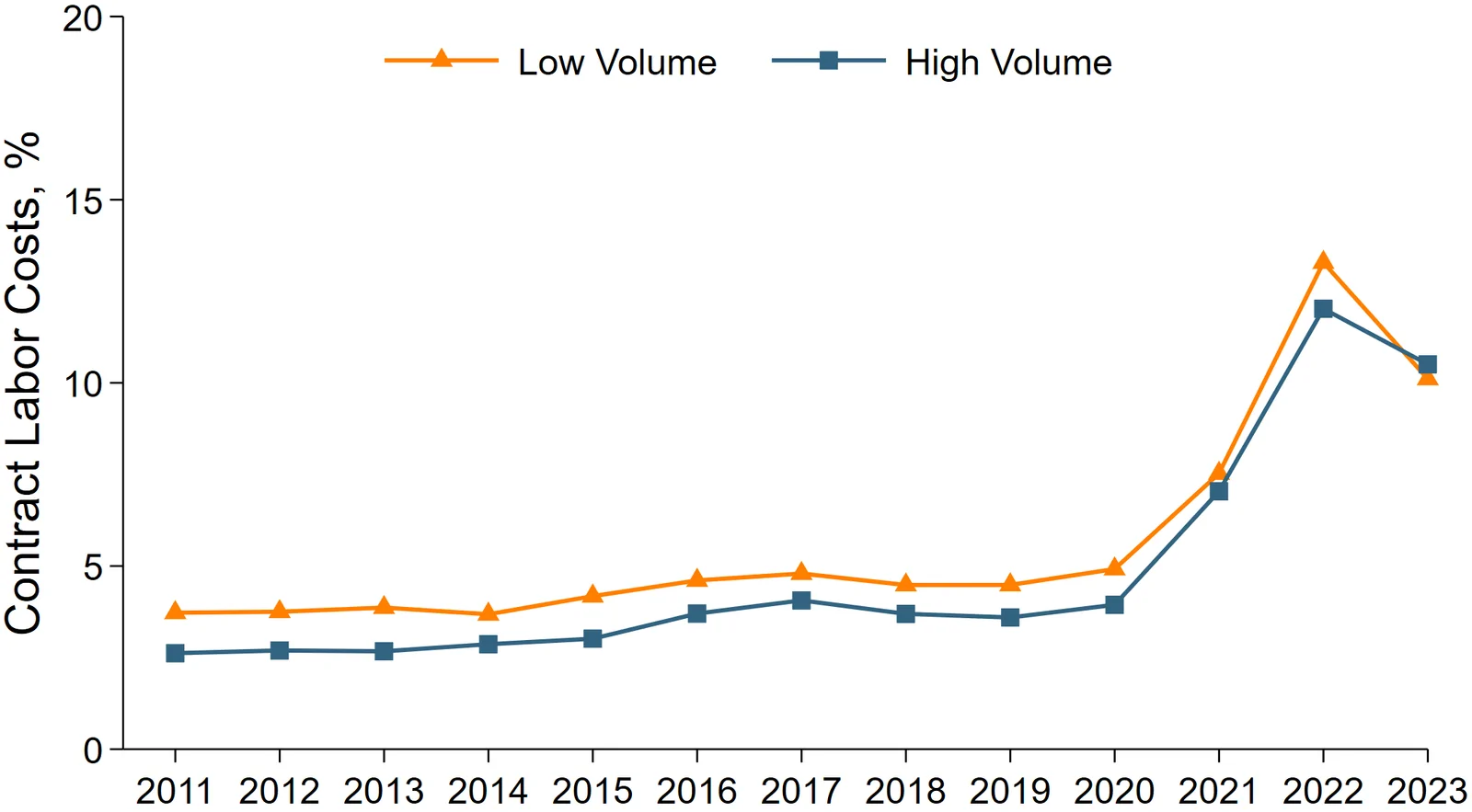
Average contract labor costs by surgical volume, 2011–2023. The figure presents the unadjusted average contract labor costs in each year for high-volume and low-volume hospitals. Contract labor costs were expressed as a percentage and defined as the direct patient care contracted labor cost divided by direct patient care labor cost.

The unadjusted mean operating margins and total margins for HVHs and LVHs for the study period are displayed in Fig 1 and Fig 2. The operating margin at HVHs and LVHs changed from 5.3% and 3.3% in 2011 to 4.7% and 2.1% in 2023. The total margin at HVHs and LVHs changed from 5.2% and 3.5% in 2011 to 6.0% and 3.0% in 2023. In each year from 2011 to 2023, the proportion of HVHs with financial distress was lower than the proportion of LVHs with financial distress (S2 Fig). From 2011 to 2023, contract labor costs increased from 2.6% to 10.5% in HVHs and from 3.7% to 10.1% in LVHs (Fig 3).

**Fig 4:**
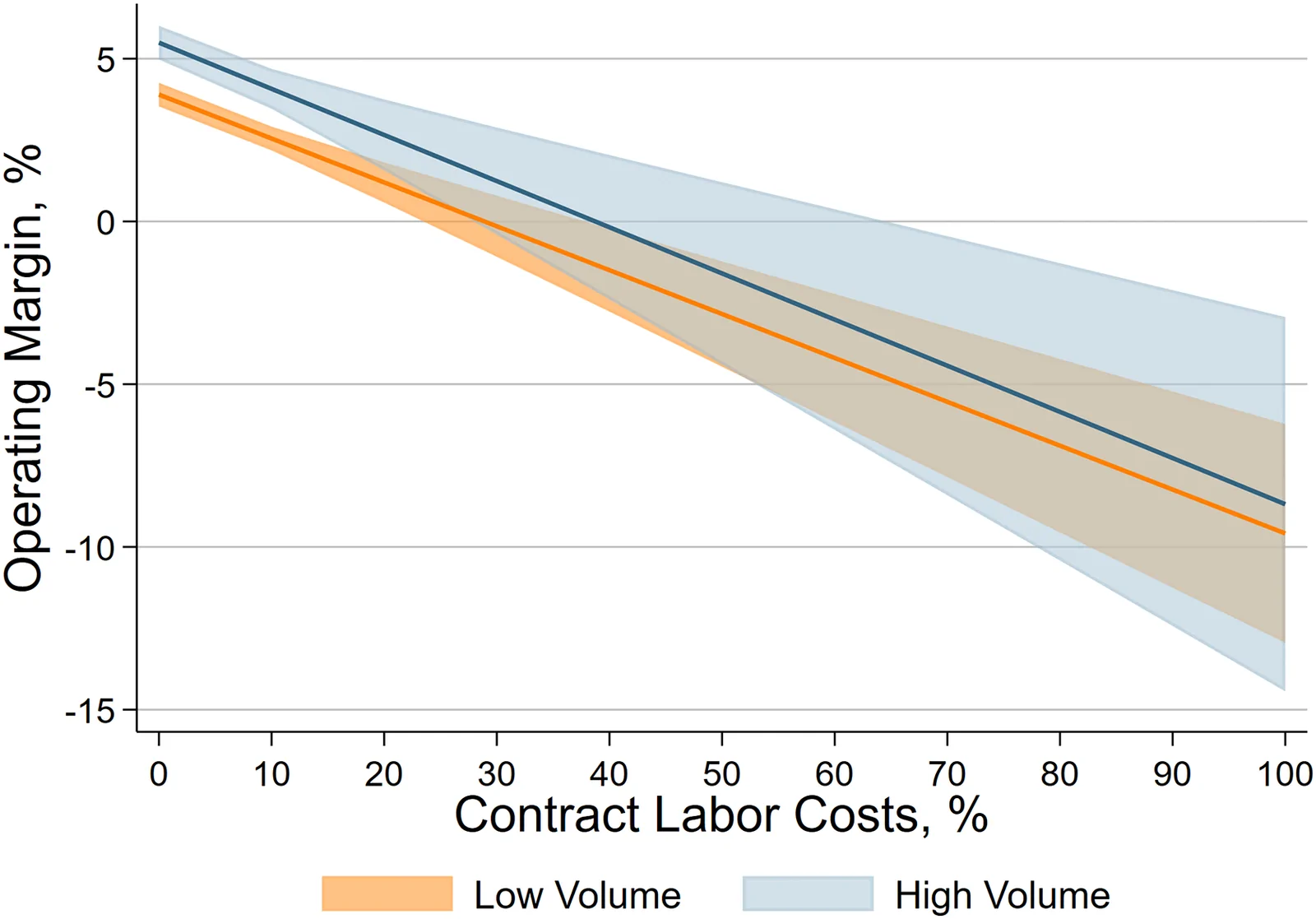
Operating margin and contract labor costs by surgical volume. Figure represents the predicted operating margin derived from the adjusted generalized estimating equation (GEE) models. Contract labor costs represent the direct patient care contracted labor costs as a percentage of direct patient care labor costs.

**Fig 5:**
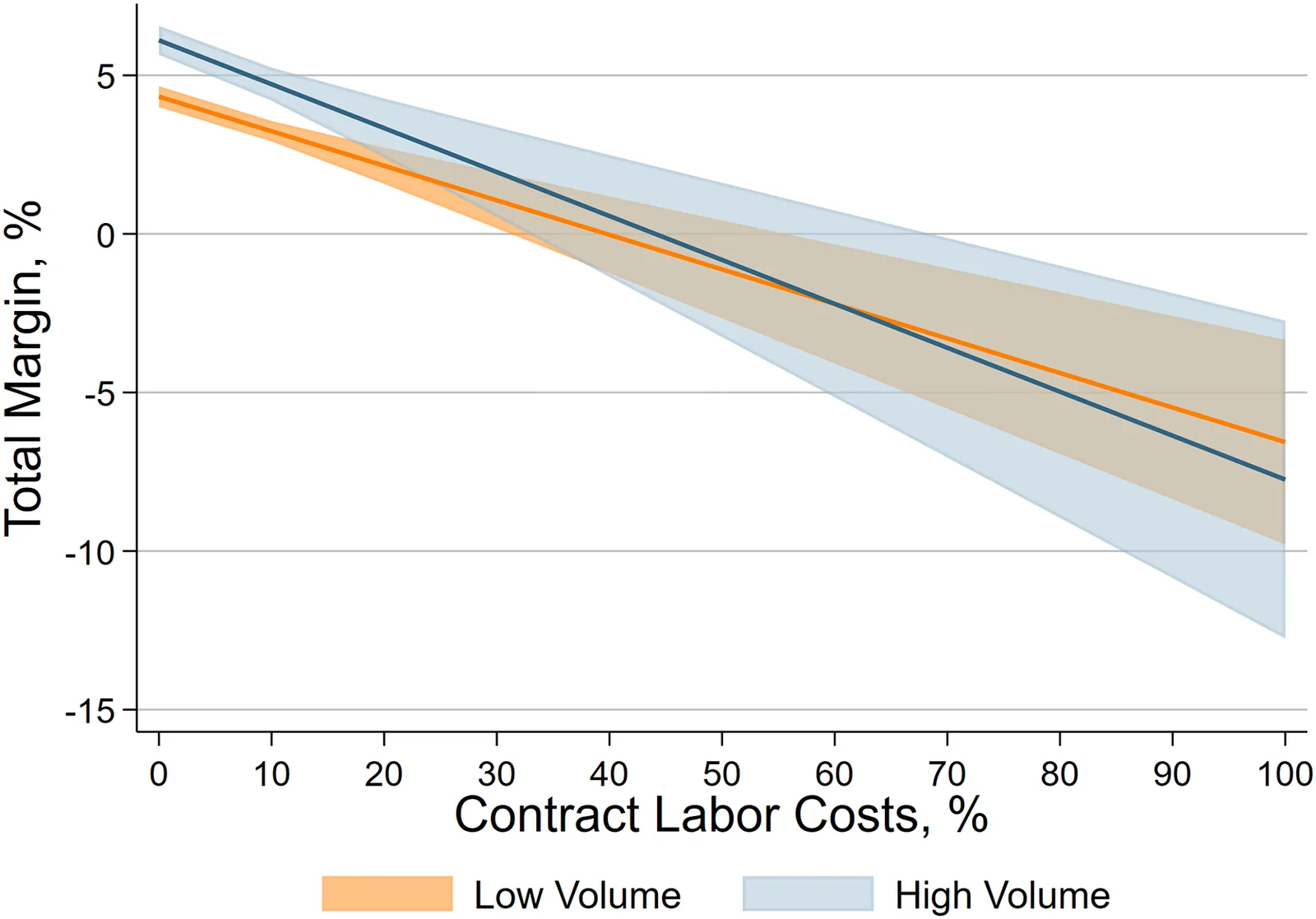
Total margin and contract labor costs by surgical volume. Figure represents the predicted total margin derived from the adjusted generalized estimating equation (GEE) models. Contract labor costs represent the direct patient care contracted labor costs as a percentage of direct patient care labor costs.

**Fig 6:**
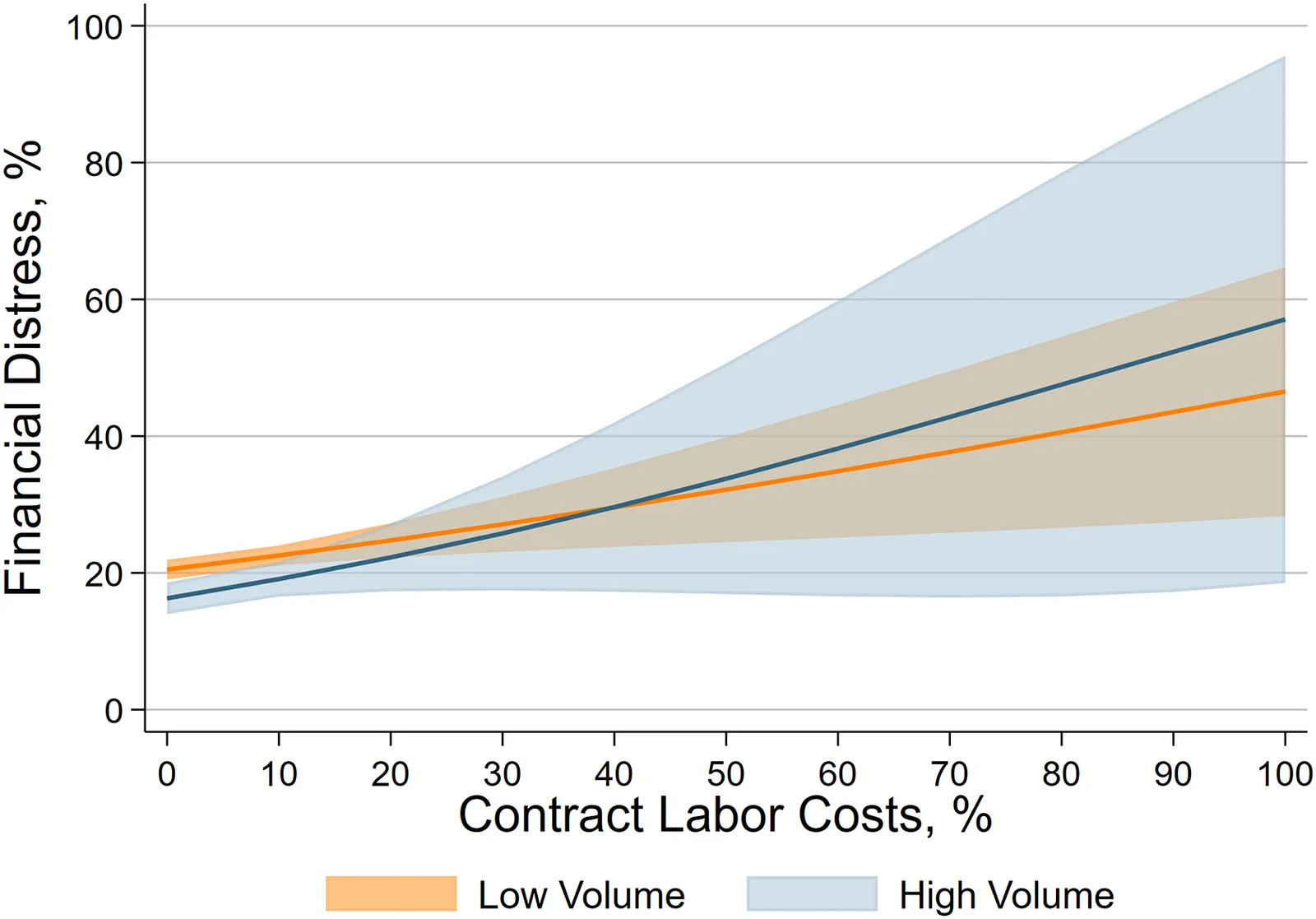
Financial distress and contract labor costs by surgical volume. The figure represents the predicted probability of financial distress based on generalized estimating equation (GEE) models, which control for rurality, teaching hospital status, bed size, ownership, system member, market concentration, wage index, and case mix index. Contract labor costs represent the direct patient care contracted labor costs as a percentage of direct patient care labor costs.

In the adjusted regression models, higher contract labor costs were associated with lower operating margins (coefficient −0.13, 95% CI −0.17 to −0.10; p < 0.001), lower total margins (coefficient −0.11, 95% CI −0.14 to −0.07; p < 0.001), and higher financial distress (odds ratio 1.01, 95% CI 1.00 to 1.02; p = 0.003; Table 2). High surgical volume was associated with higher operating margins (coefficient 1.40, 95% CI 0.86 to 1.95; p < 0.001), higher total margins (coefficient 1.62, 95% CI 1.12 to 2.12; p < 0.001), and a lower risk of financial distress (OR 0.77, 95% CI 0.64 to 0.93; p = 0.008) compared to low surgical volume. The interaction term of contract labor costs and high-volume status was not statistically significant for the models of operating margin (coefficient −0.02, 95% CI −0.08 to 0.05; p = 0.605), total margin (coefficient −0.04, 95% CI −0.09 to 0.02; p = 0.179), or financial distress (OR 1.01, 95% CI 0.99 to 1.03; p = 0.303). The regression-adjusted predicted operating and total margins for HVHs and LVHs declined as contract labor costs increased (Fig 4 and Fig 5). In addition, the predicted probability of financial distress for HVHs and LVHs increased as contract labor costs increased (Fig 6). On the sensitivity analyses with higher and lower surgical volume cutoffs, the findings were qualitatively similar (S1 Table). On the sensitivity analyses for before and after the start of the COVID-19 pandemic, our findings were qualitatively similar (S2 Table). The association between contract labor costs and operating margin was negative for both the 2011−2019 sample (coefficient −0.12, 95% CI −0.18 to −0.07; p < 0.001) and the 2020−2023 sample (coefficient −0.16, 95% CI −0.20 to −0.13; p < 0.001). Qualitatively similar findings were present for the total margin and financial distress models.

**Table 2:** Generalized estimating equation model results for associations between contract labor costs and operating margin, total margin, and financial distress.

	Operating Margin		Total Margin		Financial Distress	
Characteristic	Estimate (95% CI)	p	Estimate (95% CI)	p	Odds ratio (95% CI)	p
Contract Labor Costs	−0.133***	<.001	−0.107***	<.001	1.012***	0.003
	(−0.168 - −0.098)		(−0.141 - −0.073)		(1.004 - 1.020)	
High Volume (Ref: Low Volume)	1.404***	<.001	1.618***	<.001	0.771***	0.008
	(0.858 - 1.950)		(1.121 - 2.115)		(0.636 - 0.935)	
High Volume × Contract Labor Costs	−0.016	0.605	−0.037	0.179	1.009	0.303
	(−0.078 - 0.045)		(−0.091 - 0.017)		(0.992 - 1.027)	
Rural (Ref: Non-Rural)	−0.115	0.736	−0.172	0.591	0.597***	<.001
	(−0.781 - 0.552)		(−0.801 - 0.456)		(0.502 - 0.709)	
Teaching (Ref: Non-Teaching)	0.181	0.399	0.389**	0.049	0.904**	0.077
	(−0.239 - 0.600)		(0.002 - 0.776)		(0.809 - 1.011)	
Beds (Ref: 1–99)						
100–299	−0.089	0.783	−0.304	0.309	0.878*	0.084
	(−0.720 - 0.543)		(−0.891 - 0.282)		(0.757 - 1.018)	
> 300	−0.061	0.884	−0.216	0.577	0.679***	<.001
	(−0.884 - 0.761)		(−0.974 - 0.542)		(0.549 - 0.841)	
Ownership (Ref. Non-profit)						
Government	−2.849***	<.001	−1.479***	<.001	1.206**	0.070
	(−3.786 - −1.911)		(−2.199 - −0.759)		(0.984 - 1.476)	
For-profit	1.542***	<.001	1.156***	0.002	1.853***	<.001
	(0.767 - 2.317)		(0.439 - 1.873)		(1.532 - 2.242)	
Herfindahl Hirschman Index (Ref: Unconcentrated)						
Moderately Concentrated	0.331	0.119	0.262	0.195	0.960	0.462
	(−0.085 - 0.746)		(−0.134 - 0.657)		(0.860 - 1.071)	
Highly Concentrated	0.258	0.356	0.160	0.536	0.946	0.435
	(−0.290 - 0.807)		(−0.347 - 0.667)		(0.821 - 1.088)	
System Member	0.968***	<.001	0.413*	0.081	1.281***	0.001
	(0.441 - 1.494)		(−0.051 - 0.87)		(1.101 - 1.489)	
Wage Index	−1.540*	0.029	−0.337	0.584	1.895***	<.001
	(−2.921 - −0.158)		(−1.545 - 0.870)		(1.327 - 2.710)	
Case Mix Index	3.026***	<.001	2.349***	<.001	0.614***	<.001
	(1.945 - 4.107)		(1.354–3.344)		(0.480 - 0.785)	
Observations	22,366					

Notes: 95% confidence intervals in brackets. *** p < 0.01, ** p < 0.05, * p < 0.1. Ref = Reference group. CI = Confidence Interval

## Discussion

Our national analysis of U.S. short-term general and specialty hospitals revealed a negative association between contract labor costs and financial performance. For every one percent increase in contract labor costs as a proportion of total direct patient care labor costs, hospital operating margin declined by 0.13% and total margin decreased by 0.11%. Additionally, high surgical volume was associated with a lower risk of financial distress compared to low surgical volume. However, for both HVHs and LVHs, the predicted probability of hospital financial distress increased with the rise in contract labor costs. Collectively, our findings provide additional granularity to the ongoing conversation about the costs of healthcare delivery and the impact of rising hospital expenses, particularly temporary staff. To our knowledge, this is the first national study to examine the association between contract labor costs and financial performance at high- and low-volume hospitals.

Our findings are consistent with reports and literature regarding the cost of contract labor and its impact on hospital financial performance. In an analysis of 3000 U.S. hospitals from 2017 to 2021, the average annual hospital contract labor costs increased from $3.9 million to $7.6 million [23]. Smaller hospitals (less than 25 beds) saw an average increase of approximately $700,000 while large hospitals (greater than 250 beds) witnessed an average rise of more than $18 million. From 2019 to 2022, contract labor costs as a percent of total expenses rose from 2% to 11% [24]. Despite the growing importance of contract labor costs, literature regarding the association between contract labor costs and financial performance is limited. A previous study at one academic hospital identified a 165% rise in their health system’s nursing contract labor and an 11% decline in their surgical department’s profit margin [25]. A separate single year cross-sectional study identified a positive relationship between contract labor costs and both operating revenue and operating expenses [26]. However, this study did not explore profit margins, which aid organizational leaders in determining which activities help or hinder profitability. Our study is unique because it quantifies the association between contract labor costs and financial performance across multiple measures and uses more than a decade of financial information from a geographically expansive and diverse group of hospitals. Our sensitivity analyses also suggest that the negative association between contract labor costs and profit margins was more pronounced after the start of the COVID-19 pandemic (2020–2023) compared to prior years (2011–2019).

Our findings offer important insights for hospital leaders and policymakers. Contract labor remains a challenge for hospital and health systems across the U.S. In 2022, while contract labor nurses accounted for 23% of nursing hours, they accounted for approximately 40% of nursing labor expenses [27]. Our results provide a tangible and quantifiable measure of the impact of hiring temporary labor to fill workforce needs. While use of contract labor may address a short-term need in terms of coverage gaps, previous literature highlights the potential negative consequences (e.g., increased mortality) associated with the use of temporary labor in healthcare settings [28]. Our results add further insight into the impact related to the use of contract labor. Diversity of revenue streams and management of expenses drive organizational profitability. Given the magnitude of the association of contract labor costs on financial margins and the risk of distress, our findings highlight the importance of cost containment in navigating a hospital’s financial future. Hospitals are in a challenging situation. Insufficient staffing of nurses and physicians is associated with worse patient outcomes, and inadequate provider-to-patient ratios are linked to an increased mortality risk [29,30]. While hospitals recognize that reducing the risk of patient harm or death may require substantial financial outlays, not every hospital is in the economic position to make the investment. Medicare reimbursement continues to decline, and Medicaid revenue is at risk [31,32] Amid the persistent challenges since the onset of the COVID-19 pandemic, policymakers may need to consider legislation to bolster, not hinder, hospitals’ financial positions.

In our analysis, we did not identify any significance in the interaction of high-volume status and contract labor costs, suggesting that high volume hospitals may not have a greater ability than low volume hospitals to mitigate the potential negative impact of contract labor costs. Previous literature identified a positive association between surgical services and operating margin [33,34] However, the unique pressures of the COVID-19 pandemic and recent labor market changes may have created conditions that negatively impact overall profitability or counterbalance any efficiency gains that may be obtained at higher volume facilities. For a hospital to deliver higher surgical volume, it needs to develop greater efficiency in care delivery with its existing resources or increase its staff and providers. Greater hospital efficiency is associated with improved profitability [35]. However, the time, resources, and process to achieve greater efficiency is not often evident. Therefore, hospitals turn to hiring additional labor, and the pandemic-era market dynamics have substantially increased prices in the labor market, particularly for contract labor. Despite hospitals emerging from the pandemic era, the influence of the COVID-19 pandemic may have lasting effects on the hospital labor market.

Our study has limitations. First, selection bias may limit the generalizability of our findings. The data is retrospectively collected and reported by hospitals to the Medicare Cost Reports, and data is unavailable for a subset of U.S. hospitals. Additionally, of the hospitals that reported data, some facilities had missing values for variables used in our study. Importantly, the AHA, NASHP, and RAND datasets have been used in multiple previous analyses focused on hospital financial performance [36–39]. Second, the definition of a surgical operation may vary across reporting hospitals. The AHA does not provide a granular level of detail for this variable, and heterogeneity in the types of procedures may exist across hospitals. Our sensitivity analyses with a higher and lower cut off for surgical volume revealed qualitatively similar results for the association between contract labor costs and the financial metric of interest. The interaction term of high-volume multiplied by contract labor costs was not significant in the main analysis or sensitivity analyses, further confirming the robustness of our findings. Case mix index was included in our model to incorporate varying patient complexity between hospitals, but we acknowledge that this may not account for variation in surgical case complexity. Third, contract labor costs represented a proportion of direct patient care labor costs, and the denominator may vary in its definition across hospitals. Overall, the rising unadjusted contract labor costs trends over time are consistent with previous reports [7,40]. Fourth, there may be unobserved factors that are unaccounted for in our model. Our models adjusted for multiple hospital characteristics, fixed year effects, and the nested structure of observations within hospitals. Also, our findings were robust to separate time frames before and after the start of the COVID-19 pandemic. Nevertheless, there may be local policies or other unobserved characteristics that could bias our results.

## Conclusions

This national analysis of U.S. general and specialty hospitals revealed a negative association between contract labor costs and hospital profit margins. Hospitals with higher contract labor costs demonstrated a marginally higher risk of financial distress. High-volume surgical hospitals had higher baseline financial margins compared to low-volume hospitals, but the negative association between contract labor costs and financial performance persisted in both settings. Future state and federal policies should address the ongoing hospital reimbursement challenges amid rising labor expenses.

## Supporting information

S1 Fig Flow chart of dataset creation and exclusions. Financial variables include: Total current assets, total current liabilities, total assets, total liabilities, operating expenses, other all expenses; Key variables include: Operating margins, total margins, total current assets, total current liabilities, total assets, total liabilities, total fund balances, operating revenues, operating expenses, income from contributions, investments, and government appropriations, contract labor costs, rurality, teaching status, bed size, hospital ownership, Herfindahl-Hirschman Index, wage index, and case mix index. NASHP = National Academy for State Health Policy; AHA = American Hospital Association.
(DOCX)

S1 Appendix Description of Altman Z-score and regression model. (DOCX)

S2 Fig Financial distress by surgical volume, 2011–2023. The figure displays the proportion of hospitals experiencing financial distress in each year. A chi-square test was performed to compare the proportion of facilities in financial distress in high versus low volume hospitals for each year. The p value was statistically significant (less than 0.01) for each year.
(DOCX)

S1 Table Sensitivity analyses for the models of contract labor costs and financial performance: high volume cutoffs. 95% confidence intervals in brackets. *** < 0.01, **p < 0.05, * p < 0.10.
(DOCX)

S2 Table Sensitivity analyses for the models of contract labor costs and financial performance: 2011–2019 vs. 2020–2023. 95% confidence intervals in brackets. *** < 0.01, **p < 0.05, * p < 0.10.
(DOCX)
