# Supplementary material for: Contract labor costs and financial performance at high and low surgical volume hospitals
Source: PLoS One. 2026 Aug 27;21(8):e0356994. doi: 10.1371/journal.pone.0356994 (PMC13521374; doi:10.1371/journal.pone.0356994)
Supplement: Supplementary file 1 [file pone.0356994.s001.docx]

**S1 Fig. Flow Chart of Dataset Creation and Exclusions**

Notes: Financial variables include: Total current assets, total current liabilities, total assets, total liabilities, operating expenses, other all expenses; Key variables include: Operating margins, total margins, total current assets, total current liabilities, total assets, total liabilities, total fund balances, operating revenues, operating expenses, income from contributions, investments, and government appropriations, contract labor costs, rurality, teaching status, bed size, hospital ownership, Herfindahl-Hirschman Index, wage index, and case mix index.

NASHP = National Academy for State Health Policy; AHA = American Hospital Association
