# Supplementary material for: Contract labor costs and financial performance at high and low surgical volume hospitals
Source: PLoS One. 2026 Aug 27;21(8):e0356994. doi: 10.1371/journal.pone.0356994 (PMC13521374; doi:10.1371/journal.pone.0356994)
Supplement: Supplementary file 2 [file pone.0356994.s002.docx]

**S1 Appendix**

*Modified Altman Z-score*

The phrase “financial distress” can be defined in multiple ways. In general, financial distress refers to a state of financial decline for an organization that may or may not end in bankruptcy. Bankruptcy represents a legal declaration of financial failure and a process in which a debtor seeks to liquidate or re-organize and address existing obligations to creditors. Similar to previous literature on financial distress, we utilized the modified Altman Z-score.^1-4^ The modified Altman Z-score incorporates multiple aspects of financial health including liquidity, profitability, efficiency, and leverage. Puro et al. (2019) previously identified that the modified Altman Z-score was effective to predict financial distress resulting in bankruptcy.^3^ Based on their approach, we used the following formula for financial distress:

$$Z=6.56 \times X1+ 3.26 \times X2+ 6.72\times X3+ 1.05\times X4$$

where X1 = working capital / total assets, X2 = retained earnings / total assets, X3 = earnings before interest and taxes / total assets, and X4 = total equity / total liabilities. Given the available financial information in our datasets, we used total fund balances (total equity) as the numerator in X2 and X4. Based on Puro et al. (2019), a hospital was defined as financially distressed if its Z-score was less than 1.80.^3^

*Generalized Estimating Equation*

For our generalized estimating equation regression models, we used Stata’s xtgee command. Our dependent variables were operating profit margin, total margin, and financial distress. Our independent variables included contract labor costs, surgical volume (high vs. low), rural, teaching hospital, beds, ownership, system membership, Herfindahl-Hirschman Index, wage index, and case mix index. For the GEE models, we estimated the following equation:

$$g\left( E\left( Y_{ij} \right) \right)= {\beta_{0}+\beta}_{1}\times Contract labor costs_{ij}+\beta_{2}\times High volume_{ij}+\beta_{3}\times Contract labor costs_{ij} \times High volume_{ij}+ \text{β}_{\text{4}}\text{X}_{\text{ij}}+\gamma_{i}$$

$Y_{ij}$ includes: ${Operating Profit Margin}_{ij}$ represents the operating margin for the hospital-year $i$ within hospital $j$

$X_{ij}$ list includes: rural; teaching status; beds; ownership; market competition; wage index; system membership, and case mix index

Interaction term: high surgical volume (>=75^th^ pct) x Contract Labor Costs

We included fixed year effects ($\gamma_{i}$) and residual error term ($\epsilon_{ij}$).

$\beta_{1}$ is contract labor costs effect on the outcomes for low volume hospitals

$\beta_{1}+\beta_{3}$ is contract labor costs effect on the outcomes for high volume hospitals

$\beta_{3}$ indicates how the effect of contract labor proportion differs between high- and low-volume hospitals.

$\gamma_{i}$: random hospital effect

$g\left( \cdot\right)$: Link function

**References**

1.	Enumah SJ, Chang DC. Predictors of financial distress among private US hospitals. *Journal of Surgical Research*. 2021;267:251-259.

2.	Langabeer JR, Lalani KH, Champagne-Langabeer T, Helton JR. Predicting financial distress in acute care hospitals. *Hospital Topics*. 2018;96(3):75-79.

3.	Puro N, Borkowski N, Hearld L, Carroll N, Byrd J, Smith D, Ghiasi A. Financial distress and bankruptcy prediction: A comparison of three financial distress prediction models in acute care hospitals. *Journal of Health Care Finance*. 2019;

4.	Ramamonjiarivelo Z, Weech-Maldonado R, Hearld L, Menachemi N, Epané JP, O’Connor S. Public hospitals in financial distress: is privatization a strategic choice? *Health Care Management Review*. 2015;40(4):337-347.
