# Supplementary material for: Contract labor costs and financial performance at high and low surgical volume hospitals
Source: PLoS One. 2026 Aug 27;21(8):e0356994. doi: 10.1371/journal.pone.0356994 (PMC13521374; doi:10.1371/journal.pone.0356994)
Supplement: Supplementary file 3 [file pone.0356994.s003.docx]

**S2 Fig. Financial Distress by Surgical Volume, 2011-2023**

Note: The figure displays the proportion of hospitals experiencing financial distress each year. A chi-square test was performed to compare the proportion of facilities in financial distress in high versus low volume hospitals for each year. The p value was statistically significant (less than 0.01) for each year.
