# Supplementary material for: Contract labor costs and financial performance at high and low surgical volume hospitals
Source: PLoS One. 2026 Aug 27;21(8):e0356994. doi: 10.1371/journal.pone.0356994 (PMC13521374; doi:10.1371/journal.pone.0356994)
Supplement: Supplementary file 4 [file pone.0356994.s004.docx]

**S****1 Table. Sensitivity Analyses for the Models of Contract Labor Costs and Financial Performance: High Volume Cutoffs**

|  | **Operating Margin** | | **Total Margin** | | **Financial Distress** | |
| --- | --- | --- | --- | --- | --- | --- |
| Characteristic | Estimate (95% CI) | p | Estimate (95% CI) | p | Odds ratio (95% CI) | p |
| **High volume cutoff (70%)** |  |  |  |  |  |  |
| %Contract Labor Costs | -0.132*** | <.001 | -0.106*** | <.001 | 1.012*** | 0.003 |
|  | (-0.168 - -0.096) |  | (-0.141 - -0.072) |  | (1.004 - 1.021) |  |
| High Volume (ref: Low Volume) | 1.801*** | <.001 | 1.956*** | <.001 | 0.705*** | <.001 |
|  | (1.255 - 2.348) |  | (1.452 - 2.460) |  | (0.593 - 0.837) |  |
| High Volume × % Contract Labor Costs | -0.014 | 0.629 | -0.033 | 0.211 | 1.006 | 0.503 |
|  | (-0.072 - 0.043) |  | (-0.085 - 0.019) |  | (0.989 - 1.023) |  |
| **High volume cutoff (80%)** |  |  |  |  |  |  |
| %Contract Labor Costs | -0.133*** | <.001 | -0.110*** | <.001 | 1.014*** | 0.001 |
|  | (-0.168 - -0.098) |  | (-0.144 - -0.077) |  | (1.005 - 1.022) |  |
| High Volume (ref: Low Volume) | 1.626*** | <.001 | 1.765*** | <.001 | 0.869 | 0.203 |
|  | (1.061 - 2.190) |  | (1.232 - 2.299) |  | (0.700 - 1.079) |  |
| High Volume × % Contract Labor Costs | -0.029 | 0.392 | -0.035 | 0.244 | 1.004 | 0.689 |
|  | (-0.094 - 0.037) |  | (-0.093 - 0.024) |  | (0.985 - 1.023) |  |
| Observations | 22,366 | | | | | |

Notes: 95% confidence intervals in brackets. *** p < 0.01, ** p < 0.05, * p < 0.10.
