# Supplementary material for: Contract labor costs and financial performance at high and low surgical volume hospitals
Source: PLoS One. 2026 Aug 27;21(8):e0356994. doi: 10.1371/journal.pone.0356994 (PMC13521374; doi:10.1371/journal.pone.0356994)
Supplement: Supplementary file 5 [file pone.0356994.s005.docx]

**S2 Table. Sensitivity Analyses for the Models of Contract Labor Costs and Financial Performance: 2011-2019 vs. 2020-2023**

|  | **Operating Margin** | | **Total Margin** | | **Financial Distress** | |
| --- | --- | --- | --- | --- | --- | --- |
| Characteristic | Estimate (95% CI) | p | Estimate (95% CI) | p | Odds ratio (95% CI) | p |
| **2011-2019** |  |  |  |  |  |  |
| %Contract Labor Costs | -0.122*** | <.001 | -0.083*** | 0.001 | 1.010* | 0.080 |
|  | (-0.175 - -0.069) |  | (-0.130 - -0.036) |  | (0.999 - 1.021) |  |
| High Volume (ref: Low Volume) | 1.583*** | <.001 | 1.688*** | <.001 | 0.668*** | 0.001 |
|  | (0.997 - 2.168) |  | (1.159 - 2.218) |  | (0.532 - 0.839) |  |
| High Volume × % Contract Labor Costs | -0.014 | 0.812 | -0.018 | 0.698 | 1.027 | 0.127 |
|  | (-0.132 - 0.103) |  | (-0.111 - 0.074) |  | (0.992 - 1.064) |  |
| Observations | 16,531 | | | | | |
|  | | | | | | |
| **2020-2023** |  |  |  |  |  |  |
| %Contract Labor Costs | -0.163*** | <.001 | -0.148*** | <.001 | 1.015*** | 0.001 |
|  | (-0.200 - -0.126) |  | (-0.187 - -0.109) |  | (1.006 - 1.025) |  |
| High Volume (ref: Low Volume) | 2.174*** | <.001 | 1.834*** | <.001 | 0.708** | 0.023 |
|  | (1.262 - 3.085) |  | (0.935 - 2.733) |  | (0.525 - 0.953) |  |
| High Volume × % Contract Labor Costs | -0.0001 | 0.996 | -0.006 | 0.839 | 0.992 | 0.333 |
|  | (-0.060 - 0.060) |  | (-0.066 - 0.053) |  | (0.976 - 1.008) |  |
| Observations | 5,835 | | | | | |

Notes: 95% confidence intervals in brackets. *** p < 0.01, ** p < 0.05, * p < 0.10.
