# Supplementary material for: Contract labor costs and financial performance at high and low surgical volume hospitals
Source: PLoS One. 2026 Aug 27;21(8):e0356994. doi: 10.1371/journal.pone.0356994 (PMC13521374; doi:10.1371/journal.pone.0356994)
Supplement: Supplementary file 6 [file pone.0356994.s007.docx]

**Response to Reviewers**

**Title:** Contract labor costs and financial performance at high and low surgical volume hospitals

We thank the Reviewers for their feedback on our manuscript and for the opportunity to resubmit our manuscript. We have included their comments below with responses to each reviewer in red.

We describe the changes to the manuscript and figures. We appreciate the insightful comments provided by the Reviewers, and we believe their feedback enhances the quality of our manuscript.

**Reviewer #1**: This is an interesting article looking at the effect of labour costs on teh financial health of US hospitals. Surprisingly although there was a tendency for high volume hospitals to perform better financially this was not statistically significant. The issue of increasing casual Labour costs is a worldwide problem and is not restricted to the US so the results may be more widely applicable.

It would be helpful to look at variation in surgical procedure costs between hospitals as well as ICD codes. I believe this article is a useful addition to the literature.

Thank you for this feedback. While we do not have the granular level of detail in this study to evaluate variation in the specific surgical procedure costs between hospitals or among different ICD codes, we agree that future work exploring this concept would be helpful. This is an area of potential future inquiry.

**Reviewer #2**: This manuscript investigates the impact of contract labor costs on the financial stability of short-term general and specialty U.S. hospitals from 2011 to 2023, while evaluating whether hospital surgical volume moderates this relationship. The study is highly topical, methodologically sound, and uses an impressive longitudinal dataset, but several key sections require clarification and deeper contextualization.

The study spans from 2011 to 2023, a period that includes the major structural shock of the COVID-19 pandemic. As shown in Figure 2, contract labor costs remained relatively flat until a massive spike around 2021–2022. The authors should explicitly test or discuss whether their GEE findings are entirely driven by the pandemic-era data or if the negative relationship holds true in the pre-pandemic era (2011–2019). Running a sensitivity analysis splitting the dataset into pre-pandemic and post-pandemic periods would significantly strengthen the robustness of the findings.

Thank you for this comment and suggestion. We added a sensitivity analysis (S1 Table B). Findings are robust in both time periods. Interestingly, the profit margin coefficients are of greater magnitude in the more recent 2020-2023 sample. We report our findings in the Results section and add language to the Discussion to comment on this difference.

High-volume hospitals are classified using annual surgical volume at or above the 75th percentile. However, as noted in the limitations, the types of procedures within this volume are highly heterogeneous. A hospital performing thousands of low-margin outpatient surgeries may have a vey different financial profile than one performing complex inpatient operations. The authors should elaborate on how this lack of granularity might impact their interaction model and whether a case-mix index adjustment could help mitigate this limitation.

Thank you for this comment. We agree, and we added case mix index to our main model. Our findings remain statistically significant for our three outcomes of interest. We elaborate on the lack of granularity in the Limitations and explain that: “Case mix index was included in our model to incorporate varying patient complexity between hospitals, but we acknowledge that this may not account for variation in surgical case complexity.”

The reported odds ratio for financial distress is 1.01 (95% CI 1.01 to 1.02; p = 0.002) (p. 1). Although statistically significant due to the large sample size, an odds ratio of 1.01 suggests an incredibly small effect size per unit increase . The authors need to clarify the scaling of the independent variable in this model. Is this risk per a 1% increase in contract labor percentage, or per a different metric? If it is truly a 1% effect size, the clinical and practical significance of this specific finding should be interpreted with more caution in the discussion.

Thank you for this comment. We agree. We chose to exclude strong language from the abstract conclusion regarding financial distress and focused on the narrative on profit margins, where the findings are robust. We have added the word “marginally” to describe the higher risk of financial distress.

Minor comments:
Ensure that all references to the Supporting Information files (e.g., S1 Fig, S1 Table, S2 Table) match perfectly with the uploaded materials, as some figures are referenced in text but lack detailed data tables within the main manuscript draft.

Thank you. We included a single Supplemental File that includes all the contents (S1 Fig A, S1 File: eMethods, S1 Fig B, S1 Table A, and S1 Table B).

The abstract results section notes the exact coefficients for operating and total margins, but does not report the coefficients for the interaction terms. Adding the insignificant interaction values briefly to the abstract would provide a more complete summary of the negative hypothesis test.

Agree. We added the insignificant values to abstract.

The text indicates that American Hospital Association and RAND data cannot be shared due to data use agreements. I am unsure whether this complies fully with PLOS ONE’s data availability policy. Can the authors confirm that independent researchers can acquire these datasets through the same third-party channels?

Yes, independent researchers can acquire the American Hospital Association and RAND datasets through the same third-party channels.

**Reviewer #3**: This is an interesting and very important paper, examining (1) the association between contract labor costs and hospital financial performance, and (2) whether this relationship is modified by surgical volume. Its importance is underscored by the inflation that has been running high for the past few years across the entire world. I congratulate the authors for undertaking this important study.

The authors found that there is an association between higher contract labor costs and worse hospital financial performance, and that this relationship is not mitigated by higher surgical volume. In other words, higher surgical volume, which is generally viewed as contributing greatly to hospital margin, does not shield a hospital from higher contract labor costs.

I have some questions for clarification, to help future readers better understand this important paper:

(1) The sentences below in the introduction sets up the equipoise for the study. It is a little long. I would suggest rewriting them and shortening them to make the competing scenarios more clear:

"High-volume centers have multiple competing priorities and may need to hire temporary staff, despite remarkably high price increases, to address the clinical demand. Thus, as contract labor costs rise, high-volume surgical hospitals may be insensitive to prices and worse off compared to low-volume hospitals. However, high-volume hospitals hire contract labor to fuel profitable service lines, which include surgical care. Historically, payers reimburse more for procedural care than for non-procedural care. Thus, high-volume surgical hospitals may be somewhat shielded from the negative financial effect of rising contract labor costs as compared with low-volume hospitals."

For example, the sentence about "Historically, payers reimburse more..." may be unnecessary here.

Thank you for this comment. We agree. We re-worded sentences within this section. In addition, we removed “Historically, payers reimburse more for procedural care than for non-procedural care.”


(2) It was said the lack of mitigation by higher hospital surgical volume on the impact of higher contract labor may be due to labor market changes related to COVID (the paragraph that spans pages 13-14). However, you seem to suggest that those market dynamics are limited to "pandemic-era", but your study is on time periods after COVID, and so it is possible that the labor market changes that started during COVID may have persisted after COVID. I think you may want to edit that paragraph to highlight the possibility that COVID may have lasting effects on the labor market, even after the end of the pandemic.

Thank you for this comment. We agree. We added language to the Discussion: “Despite hospitals emerging from the pandemic era, the influence of the COVID-19 pandemic may have lasting effects on the hospital labor market.”

(3) It was said that labor costs is 56% of overall hospital expenses. Does this include all labor -- including physicians? Or are physician expenses separate because, technically, physicians are not employees of hospitals?

Yes, from the AHA’s most recent report, their analyses describe labor costs that include nurses, physicians, and other staff.

https://www.aha.org/system/files/media/file/2026/03/Costs-of-Caring-2026.pdf

(4) Can the primary multivariate analysis table be included as one of the primary figures? It is currently Supplemental Table 1, but I think it is important to see a complete multivariate table, and so it should be one of the primary tables and not a supplemental table

Yes. We agree. We now include the complete multivariable table in the main manuscript file (Table 2).

(5) I don't understand the X axis on Figures 3 and 4. Why is it "contract labor cost %" -- Why is it a %?

Thank you for this comment. In our Methods, we write that: “Our main independent variable was direct patient care contracted labor cost as a percentage of direct patient care labor cost (subsequently referred to as “contract labor costs”), and this was extracted from the NASHP dataset.”

We added clarification to the “Notes” portion of Figures 3 and 4. “Contract labor costs represent the direct patient care contracted labor costs as a percentage of direct patient care labor costs.”
