# Supplementary material for: Contract labor costs and financial performance at high and low surgical volume hospitals
Source: PLoS One. 2026 Aug 27;21(8):e0356994. doi: 10.1371/journal.pone.0356994 (PMC13521374; doi:10.1371/journal.pone.0356994)
Supplement: Peer review history

# Peer review: Decision Letter 0

**DOI:** 10.1371/journal.pone.0356994.r001

**Authors:** James Maloney

22 Jun 2026

Dear Dr. Enumah,

Please submit your revised manuscript by Aug 06 2026 11:59PM. If you will need more time than this to complete your revisions, please reply to this message or contact the journal office at plosone@plos.org. When you're ready to submit your revision, log on to https://www.editorialmanager.com/pone/ and select the 'Submissions Needing Revision' folder to locate your manuscript file.

- A letter that responds to each point raised by the academic editor and reviewer(s). You should upload this letter as a separate file labeled 'Response to Reviewers'.
- A marked-up copy of your manuscript that highlights changes made to the original version. You should upload this as a separate file labeled 'Revised Manuscript with Track Changes'.
- An unmarked version of your revised paper without tracked changes. You should upload this as a separate file labeled 'Manuscript'.

If you would like to make changes to your financial disclosure, please include your updated statement in your cover letter. Guidelines for resubmitting your figure files are available below the reviewer comments at the end of this letter.

If applicable, we recommend that you deposit your laboratory protocols in protocols.io to enhance the reproducibility of your results. Protocols.io assigns your protocol its own identifier (DOI) so that it can be cited independently in the future. For instructions see: https://journals.plos.org/plosone/s/submission-guidelines#loc-laboratory-protocols. Additionally, PLOS ONE offers an option for publishing peer-reviewed Lab Protocol articles, which describe protocols hosted on protocols.io. Read more information on sharing protocols at https://plos.org/protocols?utm_medium=editorial-email&utm_source=authorletters&utm_campaign=protocols.

As the corresponding author, your ORCID iD is verified in the submission system and will appear in the published article. PLOS supports the use of ORCID, and we encourage all coauthors to register for an ORCID iD and use it as well. Please encourage your coauthors to verify their ORCID iD within the submission system before final acceptance, as unverified ORCID iDs will not appear in the published article. *Only*  the individual author can complete the verification step; PLOS staff *cannot*  verify ORCID iDs on behalf of authors.

We look forward to receiving your revised manuscript.

Kind regards,

James P. Maloney, MD

Academic Editor

PLOS One

Journal Requirements:

When submitting your revision, we need you to address these additional requirements.

1. Please ensure that your manuscript meets PLOS ONE's style requirements, including those for file naming. The PLOS ONE style templates can be found at

https://journals.plos.org/plosone/s/file?id=wjVg/PLOSOne_formatting_sample_main_body.pdf and

https://journals.plos.org/plosone/s/file?id=ba62/PLOSOne_formatting_sample_title_authors_affiliations.pdf

2. We note that you have indicated that there are restrictions to data sharing for this study. For studies involving human research participant data or other sensitive data, we encourage authors to share de-identified or anonymized data. However, when data cannot be publicly shared for ethical reasons, we allow authors to make their data sets available upon request. For information on unacceptable data access restrictions, please see http://journals.plos.org/plosone/s/data-availability#loc-unacceptable-data-access-restrictions.

Before we proceed with your manuscript, please address the following prompts:

a) If there are ethical or legal restrictions on sharing a de-identified data set, please explain them in detail (e.g., data contain potentially identifying or sensitive patient information, data are owned by a third-party organization, etc.) and who has imposed them (e.g., a Research Ethics Committee or Institutional Review Board, etc.). Please also provide contact information for a data access committee, ethics committee, or other institutional body to which data requests may be sent.

b) If there are no restrictions, please upload the minimal anonymized data set necessary to replicate your study findings to a stable, public repository and provide us with the relevant URLs, DOIs, or accession numbers. Please see http://www.bmj.com/content/340/bmj.c181.long for guidelines on how to de-identify and prepare clinical data for publication. For a list of recommended repositories, please see https://journals.plos.org/plosone/s/recommended-repositories. You also have the option of uploading the data as Supporting Information files, but we would recommend depositing data directly to a data repository if possible.

Please update your Data Availability statement in the submission form accordingly.

3. For studies involving third-party data, we encourage authors to share any data specific to their analyses that they can legally distribute. PLOS recognizes, however, that authors may be using third-party data they do not have the rights to share. When third-party data cannot be publicly shared, authors must provide all information necessary for interested researchers to apply to gain access to the data. (https://journals.plos.org/plosone/s/data-availability#loc-acceptable-data-access-restrictions)

For any third-party data that the authors cannot legally distribute, they should include the following information in their Data Availability Statement upon submission:

1) A description of the data set and the third-party source

2) If applicable, verification of permission to use the data set

3) Confirmation of whether the authors received any special privileges in accessing the data that other researchers would not have

4) All necessary contact information others would need to apply to gain access to the data

4. If the reviewer comments include a recommendation to cite specific previously published works, please review and evaluate these publications to determine whether they are relevant and should be cited. There is no requirement to cite these works unless the editor has indicated otherwise.

**Additional Editor Comments:**

Please address the comments of the three expert reviewers. Changes are needed in some analyses in addition to other areas; these changes look feasible. Your manuscript is very interesting.

Reviewers' comments:

Reviewer's Responses to Questions

**Comments to the Author**

1. Is the manuscript technically sound, and do the data support the conclusions?

Reviewer #1: Yes

Reviewer #2: Partly

Reviewer #3: Yes

**********

2. Has the statistical analysis been performed appropriately and rigorously?

Reviewer #1: Yes

Reviewer #2: Yes

Reviewer #3: Yes

**********

3. Have the authors made all data underlying the findings in their manuscript fully available?

The PLOS Data policy

Reviewer #1: Yes

Reviewer #2: No

Reviewer #3: Yes

**********

4. Is the manuscript presented in an intelligible fashion and written in standard English?

Reviewer #1: Yes

Reviewer #2: Yes

Reviewer #3: Yes

**********

Reviewer #1: This is an interesting article looking at the effect of labour costs on teh financial health of US hospitals. Surprisingly although there was a tendency for high volume hospitals to perform better financially this was not statistically significant. The issue of increasing casual Labour costs is a worldwide problem and is not restricted to the US so the results may be more widely applicable.

It would be helpful to look at variation in surgical procedure costs between hospitals as well as ICD codes. I believe this article is a useful addition to the literature.

Reviewer #2: This manuscript investigates the impact of contract labor costs on the financial stability of short-term general and specialty U.S. hospitals from 2011 to 2023, while evaluating whether hospital surgical volume moderates this relationship. The study is highly topical, methodologically sound, and uses an impressive longitudinal dataset, but several key sections require clarification and deeper contextualization.

The study spans from 2011 to 2023, a period that includes the major structural shock of the COVID-19 pandemic. As shown in Figure 2, contract labor costs remained relatively flat until a massive spike around 2021–2022. The authors should explicitly test or discuss whether their GEE findings are entirely driven by the pandemic-era data or if the negative relationship holds true in the pre-pandemic era (2011–2019). Running a sensitivity analysis splitting the dataset into pre-pandemic and post-pandemic periods would significantly strengthen the robustness of the findings.

High-volume hospitals are classified using annual surgical volume at or above the 75th percentile. However, as noted in the limitations, the types of procedures within this volume are highly heterogeneous. A hospital performing thousands of low-margin outpatient surgeries may have a vey different financial profile than one performing complex inpatient operations. The authors should elaborate on how this lack of granularity might impact their interaction model and whether a case-mix index adjustment could help mitigate this limitation.

The reported odds ratio for financial distress is 1.01 (95% CI 1.01 to 1.02; p = 0.002) (p. 1). Although statistically significant due to the large sample size, an odds ratio of 1.01 suggests an incredibly small effect size per unit increase . The authors need to clarify the scaling of the independent variable in this model. Is this risk per a 1% increase in contract labor percentage, or per a different metric? If it is truly a 1% effect size, the clinical and practical significance of this specific finding should be interpreted with more caution in the discussion.

Minor comments:

Ensure that all references to the Supporting Information files (e.g., S1 Fig, S1 Table, S2 Table) match perfectly with the uploaded materials, as some figures are referenced in text but lack detailed data tables within the main manuscript draft.

The abstract results section notes the exact coefficients for operating and total margins, but does not report the coefficients for the interaction terms. Adding the insignificant interaction values briefly to the abstract would provide a more complete summary of the negative hypothesis test.

The text indicates that American Hospital Association and RAND data cannot be shared due to data use agreements. I am unsure whether this complies fully with PLOS ONE’s data availability policy. Can the authors confirm that independent researchers can acquire these datasets through the same third-party channels?

Reviewer #3: This is an interesting and very important paper, examining (1) the association between contract labor costs and hospital financial performance, and (2) whether this relationship is modified by surgical volume. Its importance is underscored by the inflation that has been running high for the past few years across the entire world. I congratulate the authors for undertaking this important study.

The authors found that there is an association between higher contract labor costs and worse hospital financial performance, and that this relationship is not mitigated by higher surgical volume. In other words, higher surgical volume, which is generally viewed as contributing greatly to hospital margin, does not shield a hospital from higher contract labor costs.

I have some questions for clarification, to help future readers better understand this important paper:

(1) The sentences below in the introduction sets up the equipoise for the study. It is a little long. I would suggest rewriting them and shortening them to make the competing scenarios more clear:

"High-volume centers have multiple competing priorities and may need to hire temporary staff, despite remarkably high price increases, to address the clinical demand. Thus, as contract labor costs rise, high-volume surgical hospitals may be insensitive to prices and worse off compared to low-volume hospitals. However, high-volume hospitals hire contract labor to fuel profitable service lines, which include surgical care. Historically, payers reimburse more for procedural care than for non-procedural care. Thus, high-volume surgical hospitals may be somewhat shielded from the negative financial effect of rising contract labor costs as compared with low-volume hospitals."

For example, the sentence about "Historically, payers reimburse more..." may be unnecessary here.

(1) The sentences below in the introduction sets up the equipoise for the study. It is a little long. I would suggest rewriting them and shortening them to make the competing scenarios more clear:

"High-volume centers have multiple competing priorities and may need to hire temporary staff, despite remarkably high price increases, to address the clinical demand. Thus, as contract labor costs rise, high-volume surgical hospitals may be insensitive to prices and worse off compared to low-volume hospitals. However, high-volume hospitals hire contract labor to fuel profitable service lines, which include surgical care. Historically, payers reimburse more for procedural care than for non-procedural care. Thus, high-volume surgical hospitals may be somewhat shielded from the negative financial effect of rising contract labor costs as compared with low-volume hospitals."

For example, the sentence about "Historically, payers reimburse more..." may be unnecessary here.

(2) It was said the lack of mitigation by higher hospital surgical volume on the impact of higher contract labor may be due to labor market changes related to COVID (the paragraph that spans pages 13-14). However, you seem to suggest that those market dynamics are limited to "pandemic-era", but your study is on time periods after COVID, and so it is possible that the labor market changes that started during COVID may have persisted after COVID. I think you may want to edit that paragraph to highlight the possibility that COVID may have lasting effects on the labor market, even after the end of the pandemic.

(3) It was said that labor costs is 56% of overall hospital expenses. Does this include all labor -- including physicians? Or are physician expenses separate because, technically, physicians are not employees of hospitals?

(4) Can the primary multivariate analysis table be included as one of the primary figures? It is currently Supplemental Table 1, but I think it is important to see a complete multivariate table, and so it should be one of the primary tables and not a supplemental table

(5) I don't understand the X axis on Figures 3 and 4. Why is it "contract labor cost %" -- Why is it a %?

**********

what does this mean?). If published, this will include your full peer review and any attached files.

If you choose “no”, your identity will remain anonymous but your review may still be made public.

**Do you want your identity to be public for this peer review?**  For information about this choice, including consent withdrawal, please see our Privacy Policy

Reviewer #1: No

Reviewer #2: No

Reviewer #3: No

**********

[NOTE: If reviewer comments were submitted as an attachment file, they will be attached to this email and accessible via the submission site. Please log into your account, locate the manuscript record, and check for the action link "View Attachments". If this link does not appear, there are no attachment files.]

To ensure your figures meet our technical requirements, please review our figure guidelines: https://journals.plos.org/plosone/s/figures

You may also use PLOS’s free figure tool, NAAS, to help you prepare publication quality figures: https://journals.plos.org/plosone/s/figures#loc-tools-for-figure-preparation.

NAAS will assess whether your figures meet our technical requirements by comparing each figure against our figure specifications.

# Author comment: Author response to Decision Letter 1

**DOI:** 10.1371/journal.pone.0356994.r002

7 Jul 2026

See attached "Response to Reviewers" document.

Attachment Submitted filename: Response_to_Reviewers_LaborCost_7_1.docx

# Peer review: Decision Letter 1

**DOI:** 10.1371/journal.pone.0356994.r003

**Authors:** James Maloney

12 Aug 2026

Contract labor costs and financial performance at high and low surgical volume hospitals

PONE-D-25-69202R1

Dear Dr. Enumah,

We’re pleased to inform you that your manuscript has been judged scientifically suitable for publication and will be formally accepted for publication once it meets all outstanding technical requirements.

Within one week, you’ll receive an e-mail detailing the required amendments. When these have been addressed, you’ll receive a formal acceptance letter and your manuscript will be scheduled for publication.

An invoice will be generated when your article is formally accepted. Please note, if your institution has a publishing partnership with PLOS and your article meets the relevant criteria, all or part of your publication costs will be covered. Please make sure your user information is up-to-date by logging into Editorial Manager at Editorial Manager® and clicking the ‘Update My Information' link at the top of the page. For questions related to billing, please contact billing support.

If your institution or institutions have a press office, please notify them about your upcoming paper to help maximize its impact. If they’ll be preparing press materials, please inform our press team as soon as possible -- no later than 48 hours after receiving the formal acceptance. Your manuscript will remain under strict press embargo until 2 pm Eastern Time on the date of publication. For more information, please contact onepress@plos.org.

Kind regards,

James P. Maloney, MD

Academic Editor

PLOS One

Additional Editor Comments (optional):

Very nice work on this revision.

Reviewers' comments:

Reviewer's Responses to Questions

**Comments to the Author**

Reviewer #2: All comments have been addressed

**********

2. Is the manuscript technically sound, and do the data support the conclusions?

Reviewer #2: Yes

**********

3. Has the statistical analysis been performed appropriately and rigorously?

Reviewer #2: Yes

**********

4. Have the authors made all data underlying the findings in their manuscript fully available?

The PLOS Data policy

Reviewer #2: Yes

**********

5. Is the manuscript presented in an intelligible fashion and written in standard English?

Reviewer #2: Yes

**********

Reviewer #2: The authors have addressed all of my comments - I have nothing further to suggest. I recommend publication.

**********

what does this mean?). If published, this will include your full peer review and any attached files.

If you choose “no”, your identity will remain anonymous but your review may still be made public.

**Do you want your identity to be public for this peer review?**  For information about this choice, including consent withdrawal, please see our Privacy Policy

Reviewer #2: No

**********

# Editor report: Acceptance letter

**DOI:** 10.1371/journal.pone.0356994.r004

**Authors:** James Maloney

PONE-D-25-69202R1

PLOS One

Dear Dr. Enumah,

I'm pleased to inform you that your manuscript has been deemed suitable for publication in PLOS One. Congratulations! Your manuscript is now being handed over to our production team.

At this stage, our production department will prepare your paper for publication. This includes ensuring the following:

* All references, tables, and figures are properly cited

* All relevant supporting information is included in the manuscript submission,

* There are no issues that prevent the paper from being properly typeset

You will receive further instructions from the production team, including instructions on how to review your proof when it is ready. Please keep in mind that we are working through a large volume of accepted articles, so please give us a few days to review your paper and let you know the next and final steps.

Lastly, if your institution or institutions have a press office, please let them know about your upcoming paper now to help maximize its impact. If they'll be preparing press materials, please inform our press team within the next 48 hours. Your manuscript will remain under strict press embargo until 2 pm Eastern Time on the date of publication. For more information, please contact onepress@plos.org.

You will receive an invoice from PLOS for your publication fee after your manuscript has reached the completed accept phase. If you receive an email requesting payment before acceptance or for any other service, this may be a phishing scheme. Learn how to identify phishing emails and protect your accounts at https://explore.plos.org/phishing.

If we can help with anything else, please email us at customercare@plos.org.

Thank you for submitting your work to PLOS One and supporting open access.

Kind regards,

PLOS ONE Editorial Office Staff

on behalf of

Dr. James P. Maloney

Academic Editor

PLOS One
